# Spatial distribution of early red lesions is a risk factor for development of vision-threatening diabetic retinopathy

**DOI:** 10.1007/s00125-017-4424-y

**Published:** 2017-09-07

**Authors:** Giovanni Ometto, Phil Assheton, Francesco Calivá, Piotr Chudzik, Bashir Al-diri, Andrew Hunter, Toke Bek

**Affiliations:** 1 Department of Ophthalmology, Aarhus University Hospital, DK-8000 Aarhus C, Denmark; 2 Maths and Stats Help Centre, University of Lincoln, Lincoln, UK; 3 School of Computer Science, University of Lincoln, Brayford Pool, Lincoln, LN6 7TS UK

**Keywords:** Diabetic retinopathy, Haemorrhages, Imaging, Microaneurysms, Retinal lesions, Risk factors

## Abstract

**Aims/hypothesis:**

Diabetic retinopathy is characterised by morphological lesions related to disturbances in retinal blood flow. It has previously been shown that the early development of retinal lesions temporal to the fovea may predict the development of treatment-requiring diabetic maculopathy. The aim of this study was to map accurately the area where lesions could predict progression to vision-threatening retinopathy.

**Methods:**

The predictive value of the location of the earliest red lesions representing haemorrhages and/or microaneurysms was studied by comparing their occurrence in a group of individuals later developing vision-threatening diabetic retinopathy with that in a group matched with respect to diabetes type, age, sex and age of onset of diabetes mellitus who did not develop vision-threatening diabetic retinopathy during a similar observation period.

**Results:**

The probability of progression to vision-threatening diabetic retinopathy was higher in a circular area temporal to the fovea, and the occurrence of the first lesions in this area was predictive of the development of vision-threatening diabetic retinopathy. The calculated peak value showed that the risk of progression was 39.5% higher than the average. There was no significant difference in the early distribution of lesions in participants later developing diabetic maculopathy or proliferative diabetic retinopathy.

**Conclusions/interpretation:**

The location of early red lesions in diabetic retinopathy is predictive of whether or not individuals will later develop vision-threatening diabetic retinopathy. This evidence should be incorporated into risk models used to recommend control intervals in screening programmes for diabetic retinopathy.

## Introduction

Diabetic retinopathy is a frequent cause of blindness in the Western world [1]. The disease is characterised by morphological lesions related to disturbances in retinal blood flow. The lesions display a regional distribution, with hyperperfusion and breakdown of the blood–retina barrier in the macular area and capillary occlusion in the retinal periphery that stimulates new vessel formation [2]. These regional differences may be due to differences in how blood flow is disturbed in different retinal areas [3]. The initial lesions are often observed as small red dots representing microaneurysms and/or haemorrhages developing in the macular area temporal to the fovea [4].

It has previously been shown that the early development of retinal lesions in this area may predict the development of treatment-requiring diabetic maculopathy [5]. However, the predictive value was calculated for predefined areas in the fundus selected on the basis of the authors’ clinical experience. Therefore, the study was not able to exclude the fact that the development of lesions within areas other than the ones selected for the study might better predict progression to vision-threatening retinopathy. Similarly, another previous study has suggested that the regional distribution of diabetic retinopathy lesions might be included as a variable for optimising the control interval during screening for diabetic retinopathy [6], but this assumption has not been tested in detail.

Therefore, in this study the predictive value of the location of the earliest red lesions representing haemorrhages and/or microaneurysms was studied by comparing their occurrence in a group of participants later developing vision-threatening diabetic retinopathy with that in a control group matched with respect to diabetes type, age, sex and age of onset of diabetes mellitus who did not develop vision-threatening diabetic retinopathy during a similar observation period.

## Methods

### Data selection

Two groups of participants each consisting of 30 individuals were selected from the database of diabetic retinopathy at the Department of Ophthalmology, Aarhus University Hospital. This database contains clinical data from more than 125,000 examinations performed on more than 15,000 diabetic individuals since 1992.

The first group consisted of all those in whom a screening examination during 1997 and 1998 had shown no visible signs of diabetic retinopathy, and who had been followed regularly for the following at least 9 years during which either proliferative diabetic retinopathy (PDR) or diabetic maculopathy had developed. PDR was defined as preretinal new vessels originating from larger vessels at the vascular arcades and optic nerve head [7]. Treatment-requiring diabetic macular oedema was defined as hard exudates and/or retinal oedema located within a half disc diameter from the fovea, or these lesions covering an area of at least one disc diameter, some of which were located within one disc diameter of the fovea [8]. The second group consisted of participants matched pairwise with those in the first group with respect to diabetes type, age at onset and duration of diabetes mellitus, who had been followed for a period similar to the first group without developing vision-threatening diabetic retinopathy. During this observation period, at least seven examinations were performed on each individual, with an average interval of 1.57 years in the first group and 1.89 years in the second group, which amounted to a total of 449 examinations for the studied participants. None of these individuals received any pharmacological or surgical treatment for ocular disease during the follow-up period. The available baseline characteristics of the participants are shown in Table 1, and the numbers and intervals of the screening examinations in Table 2.

**Table 1:** Baseline characteristic of the study participants

Variable	Group reaching treatment endpoint	Control group
	Mean (min–max)	Mean (min–max)
Age (years)	37.9 (16.4–65.1)	36.9 (9.2–64.4)
Diabetes duration (years)	6.0 (0.02–21.9)	5.0 (0.01–22.0)

**Table 2:** Numbers and intervals of the screening examinations on the study participants

Variable	All patients	Patients with progression	Patients without progression
Screening period (years)	12.88 (1.89)	12.52 (1.96)	13.24 (1.75)
Number of visits	7.48 (2.57)	7.97 (2.48)	7.00 (2.60)
Screening interval (years)	1.72 (0.64)	1.57 (0.55)	1.89 (0.74)

Data are presented as means ± SD

At each examination, mydriasis was induced by eye drops containing tropicamide 1% (Alcon, Copenhagen, Denmark) and phenylephrine 2.5% (SAD, Danish Hospital Pharmacies, Skanderborg, Denmark), after which two photographs were taken for each eye, one centred at the optic disc and the other at the fovea. The images were recorded using a Canon CF-60UV fundus camera (Canon, Tokyo, Japan) on Kodak Ectachrome 64 colour diapositive film (Kodak, Rochester, NY, USA) before 1 March 2002 (60° field of view), and using a digital camera unit (FinePix S1 Pro; Fujifilm, Minato, Tokyo, Japan) after this date (45° field of view). All image processing procedures were performed within the area covered by the smallest field of view. A macula-centred photograph from each eye was selected for the analysis, only including photographs in focus and with sufficient illumination; this amounted to the study of photographs from 998 eyes. For the further analysis, the photographs were anonymised so that the participants were allocated a random number between 1 and 60, but so that sequential images belonging to the same individual could be identified.

### Image processing

**Fig. 1:**
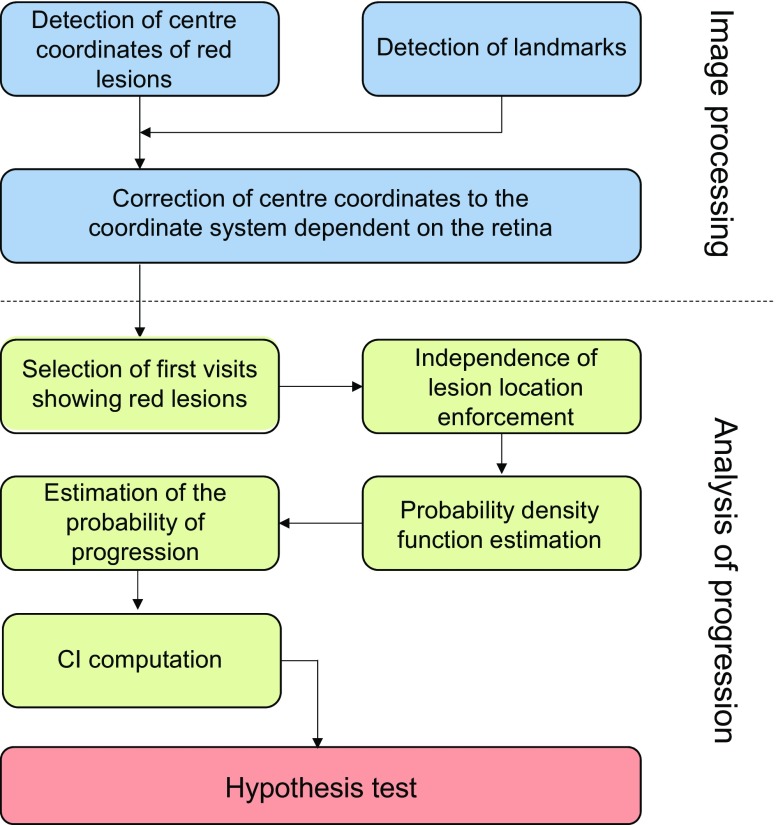
The sequence of events in the analysis

All 998 images from the two eyes were processed individually by a two-step procedure: (1) the detection of red lesions; and (2) an adjustment of the coordinates of each lesion to the individual points of reference in each retina (Fig. 1). For the image processing, scripts were written in MATLAB (version 2015a; MathWorks, Natick, MA, USA).

#### Detection of red lesions

The dataset was processed using the algorithm for the automatic detection of dark lesions presented by Hunter et al [9]. These lesions can represent either haemorrhages or microaneurysms, which cannot be differentiated on the basis of fundus photographs. Subsequently, the centre points of red lesions detected by the algorithm were displayed on the original photographs. The script allowed a correction of the identifications by single mouse clicks. All photographs in the dataset and the detected red lesions were displayed in sequence and were reviewed by the first author (G. Ometto) to select all true red dots and remove false-positive selections. The final selection of red dots in each photograph was recorded in a separate text file as a list of *x*–*y* coordinates. The procedure was repeated by a retina specialist (T. Bek) on a random selection of 5% of the images in the dataset.

#### Correction of coordinates

**Fig. 2:**
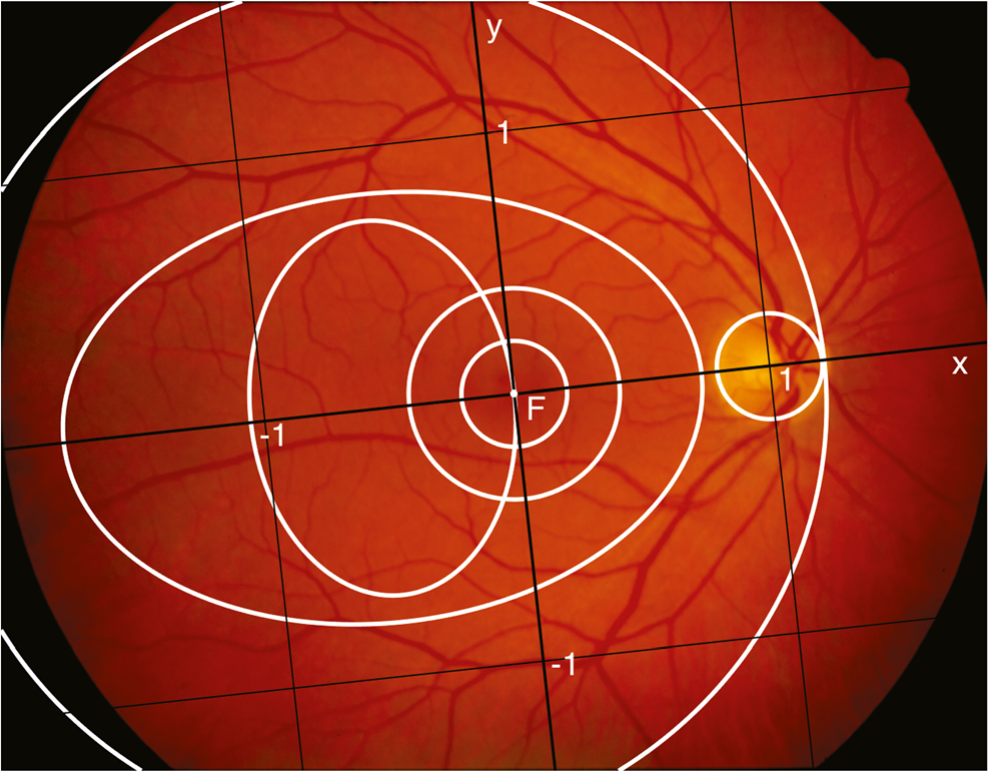
The landmark-based coordinate system (black lines) centred on the fovea (F) of a fundus photograph from the dataset. The circular and elliptical regions described by Hove et al [19] are delimited by white lines. − 1 and 1 represent the unit along the *x* and *y* axes

The automatic approach described by Ometto et al [10] was used to detect the optic disc and the fovea. An *x*-axis of a coordinate system in the fundus was identified by the line through the centres of the fovea and the optic disc, with the distance between these two structures defined as one unit of length. The *y*-axis was defined as the line orthogonal to the *x*-axis through the fovea, and the unit along this axis was defined by the average of the distances from the origin and the two points where the ellipse fitting the vascular arcades crossed this axis. This coordinate system divided the photographs into four quadrants, with the origin at the fovea (Fig. 2). The landmarks detected by the automatic lesion detection algorithm were saved in a text file.

Subsequently, a script assisted the manual review of the landmarks that had been automatically detected; this was performed by the first author (G. Ometto) and by a retina specialist (T. Bek). The coordinates were displayed on the respective photographs, where the user could change the selected coordinates with mouse clicks and move and stretch an elliptical approximation of the detected optic disc to fit with its actual boundary. Finally, the coordinates of the identified red lesions were converted to the new coordinate system using the manually reviewed landmarks of the respective photographs and were saved in a csv file.

### Analysis of progression

The corrected coordinates of the lesions were used to obtain statistical evidence about the relationship between the location of early red lesions and the risk of progression of retinopathy to a vision-threatening stage (Fig. 1). For these analyses, scripts were written in R (version R 3.1.0; Bell Labs, Murray Hill, NJ, USA).

#### Selection of the visits showing the first red lesions

The first examinations from each participant for whom at least one red lesion was detected in at least one eye were selected for the analysis. This resulted in the identification of 30 examinations from the group with progression and 23 from the group with no progression, implying that in seven individuals no retinopathy had developed during the observation period. A total of 155 and 49 red lesions were detected in the photographs from the two groups, respectively.

#### Independence of lesion locations

The occurrence of individual retinopathy lesions was considered to be independent when they were separated by more than 500 μm and therefore could be assumed to be related to different microvascular units [11]. This distance in μm was converted to a distance in pixels (*d*) by assuming that the distance in pixels between fovea and the centre of the optic disc (*d*_*F* − *OD*_) represented 4500 μm [12], according to the following:

$$ d={d}_{F- OD}\times \left(\frac{500\ \upmu m}{4500\ \upmu m}\right) $$

Subsequently, the photographs containing the first registered lesions were identified. Among the lesions with an interindividual distance smaller than *d*, all but one was removed at random. This process left 109 lesions in the first group and 39 in the second group. The coordinates of lesions occurring in the left eyes were flipped horizontally around the *y*-axis, allowing the representation of locations from both eyes in the coordinate system of the right eye. This increased the number of lesions while maintaining the assumption of each lesion belonging to a separate microcirculatory unit in the same individual.

#### Probability density function

**Fig. 3:**
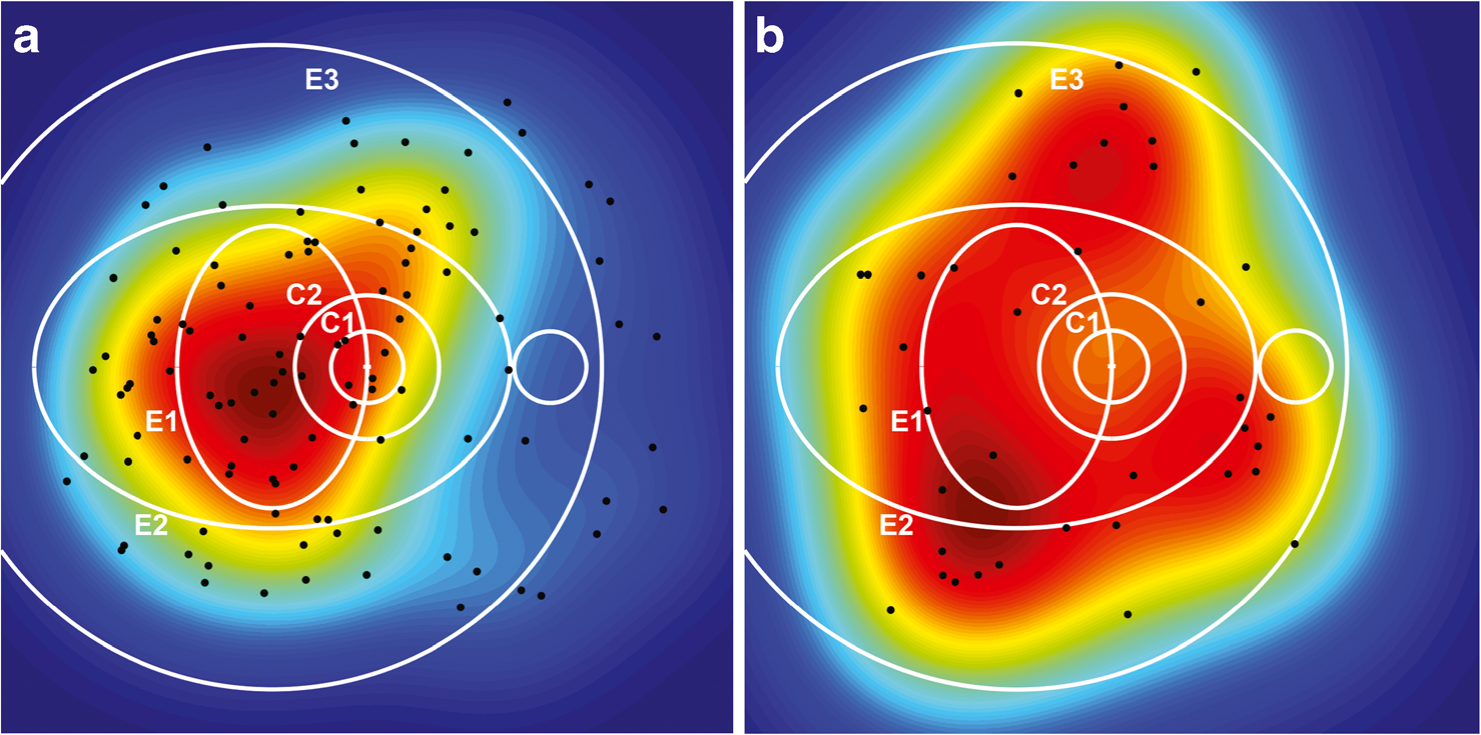
Probability density functions obtained with kernel density estimation for the locations of early red lesions from participants with (**a**) and without (**b**) progression. The colour scale ranging from blue (lowest values) to dark red (highest values) represents the surface under which the area has a total of 1 in each diagram. The black dots represent the locations of the observed red lesions. The circular (C1, C2) and elliptical (E1, E2, E3) regions described by Hove et al [19] are shown in white for reference

For each participant, the location of lesions was used to calculate the probability distribution in each point of the map (Fig. 3) using the kernel density estimation technique [13, 14]. The maps generated for all lesions within each of the two groups were averaged to obtain the probability density functions.

#### Probability of progression

The probability of the location of the first observed red lesions given progression to vision-threatening retinopathy was converted to the probability of progression to vision-threatening retinopathy given the location of the first observed red lesion using Bayes’ theorem. The probability of progression to vision-threatening retinopathy was estimated from the total number of individuals referred for either PDR or diabetic maculopathy in the screening programme from 1 April 2013 to 31 March 2016 (476) divided by the total number of examinations performed during the same period (12,329), and was equal to 0.0386.

#### Confidence interval

For each group, a new dataset was obtained by successive random selections from the whole group, until the new sample contained the same number of observations as the original sample. This sampling procedure was subsequently repeated *n* = 10,000 times for each of the two groups in accordance with the bootstrapping technique [15]. These new datasets were used to calculate *n* probabilities of progression with the procedure described for the original groups, and a 99% CI was provided from a derivation of the bias corrected and accelerated (BC_a_) bootstrap CI [16]. The CI was calculated on a 100 × 100 resolution map, which was subsequently increased by a factor of 10 using quadratic Interpolation.

#### Hypothesis testing

We tested the null hypothesis that the probability of progression to vision-threatening diabetic retinopathy was independent of the location of the first red lesion, which would be confirmed if the average probability of progression (3.86%) was within the lower and upper limits of the 99% CI at every location of the map. Rejection of the hypothesis would support the alternative hypothesis that the location of the first red lesion was a risk factor for progression.

### Subgrouping according to the type of vision-threatening retinopathy

The analyses were repeated comparing the group with diabetic maculopathy, which included 36 observations from 11 participants, with the group with PDR, which included 73 observations from 19 participants, using an average probability of progression to diabetic maculopathy equal to 0.43.

## Results

**Fig. 4:**
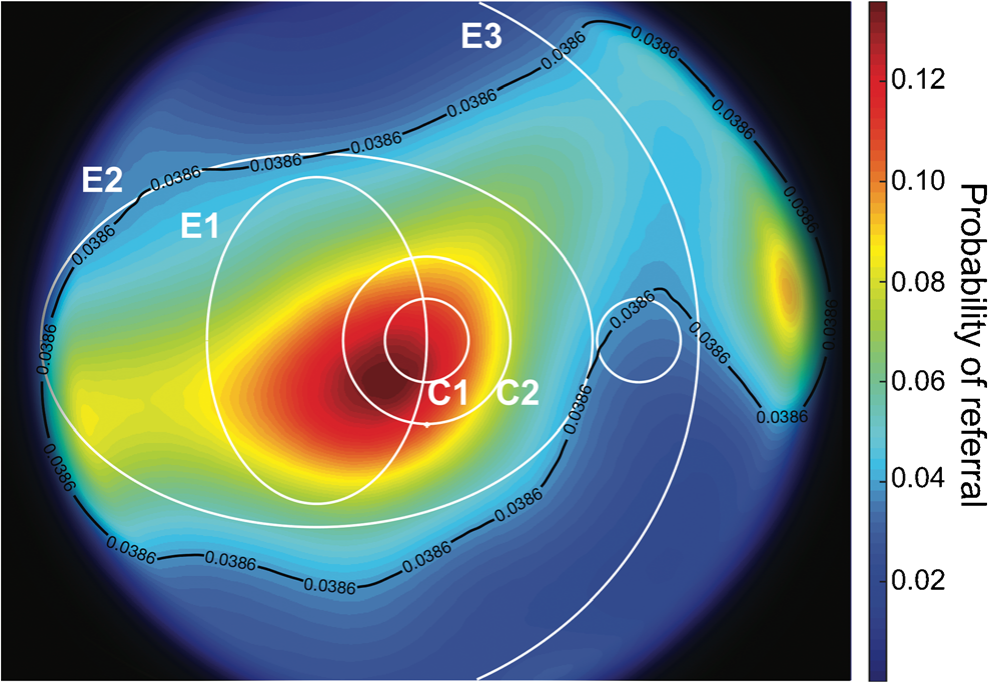
The probability of progression to vision-threatening diabetic retinopathy as a function of the location of the initial lesion. The black line represents the average probability of progression estimated from the database of the screening programme for diabetic retinopathy. The circular (C1, C2) and elliptical (E1, E2, E3) regions described by Hove et al [19] are shown in white for reference

The probability of progression to vision-threatening diabetic retinopathy is shown in Fig. 4 as a function of the location of the first red lesions observed in the dataset. The black line represents the average probability (3.86%) estimated from the database of the screening programme for diabetic retinopathy, which contains clinical data for all individuals referred for treatment in the screening programme. It appears that the risk of developing the first lesions was higher within E2, and the peak value (14.22%) was calculated for lesions located inside E1 and C2, representing a risk 3.68 times higher than the average risk of progression.

**Fig. 5:**
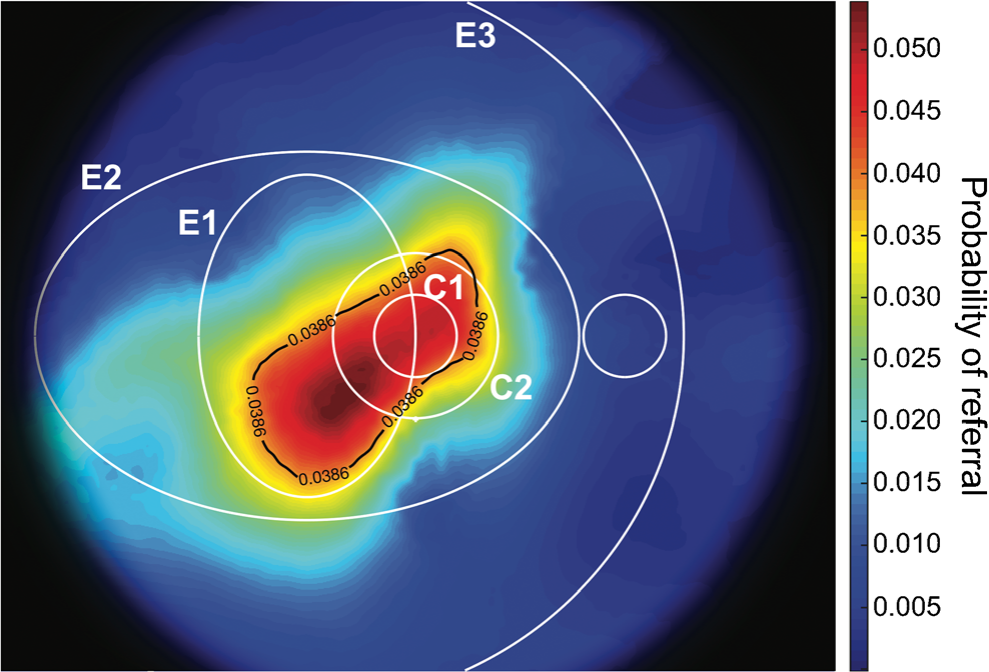
The lower limit of the risk of progression to vision-threatening diabetic retinopathy obtained with the 99% CI. The black line represents the average risk of progression and identifies the area of increased risk. The circular (C1, C2) and elliptical (E1, E2, E3) regions described by Hove et al [19] are shown in white for reference

The average risk of progression was outside the lower limit of the 99% CI of the risk of progression, implying that the location of the lesions was a risk factor for progression to vision-threatening diabetic retinopathy. The area where the development of the first red lesion represented an increased risk of developing vision-threatening diabetic retinopathy at the 99% confidence limit is shown in Fig. 5. The peak value (5.39%) represented a probability of progression that was 39.5% higher than the average. There was no significant difference in the location of the first red lesion in individuals eventually developing diabetic maculopathy or PDR.

## Discussion

The aim of the study was to investigate the predictive value of the location of the first haemorrhage/microaneurysm (red lesion) for the development of vision-threatening diabetic retinopathy. However, the detection of the first red lesion cannot be guaranteed in a screening programme with examination intervals of more than 1 year in a disease with lesions showing a turnover with development and resolution within a few weeks [17, 18]. This may be the background for the finding that the number of first detected lesions was higher in individuals progressing to vision-threatening diabetic retinopathy than in those not progressing to this retinopathy stage. However, the fact that the first detected lesions were few in number indicates that the first lesions that had actually developed had contributed significantly to the observations used to predict the prognosis. The evidence gained from the study can be expected to be extended in future studies, which will require larger sample sizes. This will allow an evaluation of the role of systemic risk factors, the size, shape and heterogeneity of individual lesions, and lesions located outside a fovea-centred fundus photograph for the pattern of development of diabetic retinopathy.

The study confirms previous clinical observations [2, 4] that the earliest diabetic retinopathy lesions develop in the macular area temporal to the fovea, as well as the results of cohort studies showing that the occurrence of lesions in this area predicts the development of diabetic maculopathy [5]. The current research extends these previous studies by showing that the development of early red lesions temporal to the fovea also predicts progression to PDR, and therefore emphasises the common background of the two vision-threatening complication types. This suggests that the risk of development of retinopathy into either of the two complication types may depend on risk factors unrelated to the occurrence of the first retinopathy lesions.

The study also extends existing evidence by providing a detailed definition of the area where the development of red lesions is accompanied by an increased risk of later progression to vision-threatening retinopathy. This area had a more circular shape than the vertically oriented ellipse temporal to the macular area selected arbitrarily in the previous study to represent the area where the earliest retinopathy lesions tend to develop [19]. The area temporal to the fovea corresponds to the area of the retinal vascular system located furthest from the larger vascular arcades, which emphasises that diabetic retinopathy is a microvascular disease. Conversely, the earliest lesions in individuals who do not show progression to vision-threatening diabetic retinopathy developed around the vascular arcades, where the arterial pressure trauma can be expected to be highest, suggesting that they may represent the consequences of impaired pressure autoregulation [3, 20–22].

The area where the occurrence of early red lesions could predict the development of vision-threatening diabetic retinopathy with 99% certainty was predominantly located temporal to the fovea, but slightly below the horizontal meridian. The location of this area was defined on the basis of the observations available for the study, and it cannot be excluded that the area would have been larger and perhaps had resembled the observed distribution of early lesions shown in Fig. 4 if the number of observations had been larger. An evaluation of this issue should be the subject of a future study based on a larger dataset.

The findings of the present study confirm that the location of early diabetic retinopathy lesions has diagnostic and prognostic value in daily clinical practice. Thus, it is of practical importance that first red lesions developing temporal to the fovea are more predictive of the development of vision-threatening diabetic retinopathy over 10 years later than first red lesions developing elsewhere. However, it is likely that the site of location of lesions outside the observed image field, their morphological characteristics, the first element of other lesions, and the pattern of development of diabetic retinopathy following the first lesions might also carry valuable diagnostic and prognostic value. This should be pursued in future extensions and improvements of the methodology.

However, even with access to all information contained in the pattern of development of diabetic retinopathy, it is not likely that this information can provide a full risk profile for the development of diabetic retinopathy. This requires integration into decision models containing other relevant risk factors such as type of diabetes, age, sex, diabetes duration, HbA_1c_ level and blood pressure [23]. Providing the basis for and integrating this information into risk models to be used in daily clinical practise can be expected to improve the management of diabetic retinopathy in future.

## Abbreviations

PDR: Proliferative diabetic retinopathy
